# Taxonomic revision of the *Pyrgulopsis gilae* (Caenogastropoda, Hydrobiidae) species complex, with descriptions of two new species from the Gila River basin, New Mexico

**DOI:** 10.3897/zookeys.429.7865

**Published:** 2014-07-30

**Authors:** Robert Hershler, Victoria Ratcliffe, Hsiu-Ping Liu, Brian Lang, Claire Hay

**Affiliations:** 1 Department of Invertebrate Zoology, Smithsonian Institution, P.O. Box 37012, Washington, DC 20013-7012, USA; 2 Department of Biology, Metropolitan State University of Denver, Denver, CO 80217, USA; 3 New Mexico Department of Game and Fish, One Wildlife Way, Santa Fe, NM 87507, USA; 4 Department of Earth and Atmospheric Sciences, Metropolitan State University of Denver, Denver, CO 80217, USA

**Keywords:** Gastropoda, United States, freshwater, taxonomy, conservation

## Abstract

We describe two new species of springsnails (genus *Pyrgulopsis*) for populations from the middle Fork and upper East Fork of the Gila River Basin (New Mexico) that had been previously identified as *P. gilae*. We also restrict *P. gilae* to its originally circumscribed geographic range which consists of a short reach of the East Fork Gila River and a single spring along the Gila River (below the East Fork confluence). These three species form genetically distinct lineages that differ from each other by 3.9–6.3% for mtCOI and 3.7–8.7% for mtNDI (the latter data were newly obtained for this study), and are diagnosable by shell and penial characters. Collectively the three species form a strongly supported clade that is distinguished from other congeners by the unique presence of two glandular strips on the dorsal surface of the penial filament. These findings suggest that the conservation status of *P. gilae*, which was recently removed from the list of candidates for listing as endangered or threatened by the United States Fish and Wildlife Service, should be revisited and that the two new species may also merit protective measures given their narrow geographic ranges.

## Introduction

*Pyrgulopsis* is a large genus (137 species; Hershler et al. 2013) of freshwater gastropods that is distributed in North America west of the Mississippi River basin. The tiny species in this genus live in spring-fed habitats and usually have very small geographic ranges. *Pyrgulopsis* is a current focus of conservation efforts owing to threats posed by groundwater extraction, livestock grazing and other anthropogenic activities (Hershler et al. 2014). Recent molecular studies have shown that several congeners are composites of genetically divergent lineages and are in need of taxonomic revision (e.g., Liu et al. 2003, Liu and Hershler 2007, Hershler and Liu 2010). This is the second in an anticipated series of papers that clarifies the taxonomy of these species (Hershler et al. 2013).

*Pyrgulopsis gilae* (Taylor, 1987) was described for specimens from single springs along the lower East Fork (type locality) and main stem Gila River in Grant County, New Mexico. Field surveys in the 1990’s and 2000’s resulted in the discovery of new populations in two other reaches of the upper Gila River watershed (Middle Fork, upper East Fork) that are currently being treated as *Pyrgulopsis gilae* (NMDGF 2012). Hurt (2004) delineated substantial divergence in mtCOI sequences (6.8% average) between specimens from the upper East Fork reach (Wall Spring) and three localities within the originally circumscribed range of *Pyrgulopsis gilae*. In a more comprehensive survey of COI variation within *Pyrgulopsis gilae*, populations from the upper East Fork, Middle Fork, and lower East Fork (and main stem Gila River) reaches were resolved as three divergent (3.9-6.3%) sub-clades which were postulated to be distinct species (Liu et al. 2013). Here we document a congruent pattern of variation in a second mitochondrial DNA marker (NDI) and delineate morphological differences supporting recognition of the upper East Fork and Middle Fork Gila River populations as new species, which are described herein.

## Methods

**Figure 1.**
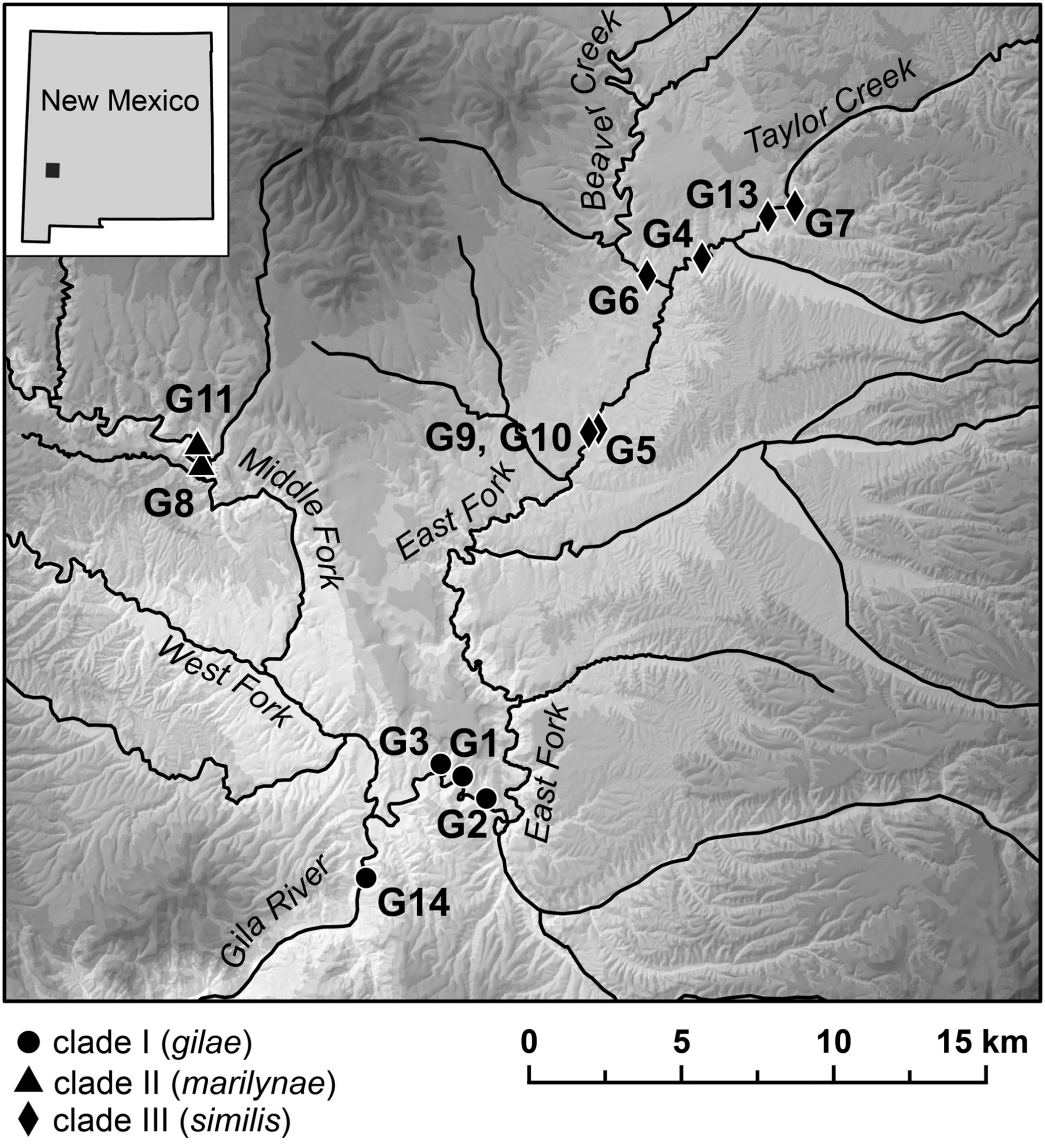
Map showing the distribution of mtDNA clades I-III.

For the current molecular study we used the same samples that were analyzed in our previous phylogeographic investigation of *Pyrgulopsis gilae* across its entire geographic range (Liu et al. 2013; Fig. 1). Genomic DNA was extracted from single, entire snails using a CTAB protocol (Bucklin 1992); 3-8 specimens were analyzed (separately) from each sample. ND43F and RND592F (Liu et al. 2003) were used to amplify a 530 bp fragment of NADH dehydrogenase subunit I (NDI). This primer pair did not amplify the region for specimens from two localities (G8, G11) and consequently we designed a second set of oligonucleotide primers for these snails, ND30 (5'TCT TAY ATR CAR ATW CGT AAA GG3') and RND490 (5'ATG TTA CAA ATC ATA TAA ATG3'), based on conserved regions of NDI in an alignment from *Pyrgulopsis gilae* and two closely related species (*Pyrgulopsis deserta* [Pilsbry], *Pyrgulopsis davisi* [Taylor]). Degenerate positions are represented by the following ambiguity codes: Y=C/T; R=A/G; W=A/T. Amplification conditions and sequencing of amplified polymerase chain reaction product followed Liu et al. (2003). Sequences were determined for both strands and then edited and aligned using Sequencher™ version 5.0.1. The 56 newly sequenced specimens of *Pyrgulopsis gilae* were analyzed both separately and together with our previously published COI dataset (Liu et al. 2013). We included the same set of outgroup taxa as in our prior study of *Pyrgulopsis gilae* (Liu et al. 2013), with *Floridobia floridana* again being used as the root. The GenBank accession numbers for these sequences (COI, NDI) are given in Appendix 1. Note that we newly sequenced specimens of *Pyrgulopsis* “*mimbres*” for COI (using the methods of Liu et al. 2003) and NDI as part of this study (GenBank accession numbers: COI, KM205358; NDI, KM205359). The newly obtained haplotypes from each *Pyrgulopsis gilae* sampling locality were deposited in GenBank. Sample information and GenBank accession numbers are given in Table 1. One example of each haplotype detected in a given sample was used in our analyses.

The partition homogeneity/incongruence length difference test (Farris et al. 1994) was used to determine whether the COI and NDI datasets were consistent and could be combined for the phylogenetic analysis. This test, which was conducted using parsimony-informative sites only and 1,000 replicates, did not detect significant incongruence (*P*=0.21) and consequently we combined the two datasets in the phylogenetic analysis. MrModeltest 2.3 (Nylander 2004) was used to obtain an appropriate substitution model (using the Akaike Information Criterion) and parameter values for this analysis. Phylogenetic relationships were inferred by Bayesian analysis using MrBayes 3.1.2 (Huelsenbeck and Ronquist 2001). Metropolis-coupled Markov chain Monte Carlo simulations were run with four chains (using the model selected through MrModeltest) for 2,000,000 generations, and Markov chains were sampled at intervals of 10 generations to obtain 200,000 sample points. We used the default settings for the priors on topologies and the GTR + I + G model parameters selected by MrModeltest as the best fit model. The Tracer program was used to analyze runs for Effective Sample Size (ESS, greater than 200) to ensure that sufficient sampling occurred. At the end of the analysis, the average standard deviation of split frequencies was less than 0.01 (0.002) and the Potential Scale Reduction Factor (PSRF) was 1, indicating that the runs had reached convergence. The sampled trees with branch lengths were used to generate a 50% majority rule consensus tree with the first 25% of the samples removed to ensure that the chain sampled a stationary portion.

Genetic relatedness within *Pyrgulopsis gilae* was further assessed by a haplotype network that was generated by TCS version 1.21 using the default settings (e.g., 95% connection limit) and fixing the connection limit at 90 steps (Clement et al. 2000). NDI sequence divergences (maximum composite likelihood) within and between lineages were calculated using MEGA6 (Tamura et al. 2013), with standard errors estimated by 1,000 bootstrap replications with pairwise deletion of missing data. Structuring of variation among lineages was evaluated by an AMOVA using Arlequin 3.5 (Excoffier and Lischer 2010).

Types and other voucher material were deposited in the National Museum of Natural History (USNM) collection. Specimens of *Pyrgulopsis gilae* from the Bell Museum of Natural History (BellMNH) were also examined during the course of this study. Series of large adults (*n*>10) were used for shell measurements. Whorl counts refer to the entire shell. Sexual dimorphism in shells, which is occasionally observed in *Pyrgulopsis* (Taylor 1987), could not be quantified owing to small sample sizes. The total number of shell whorls was counted (WH) for each specimen; and the height and width of the entire shell (SH, SW), body whorl (HBW, WBW), and aperture (AH, AW) were measured from camera lucida outline drawings using a digitizing pad linked to a personal computer (see Hershler 1989). In addition, three ratios were generated from the raw data (SW/SH, HBW/SH, AH/SH). Descriptive statistics were generated using Systat for Windows 11.00.01 (SSI 2004). T-tests (two-tailed) of differences among shell variables were conducted using an on-line calculator (http://in-silico.net/tools/statistics/ttest); data for type material of *Pyrgulopsis gilae* were from Taylor (1987, table 11). Penial variation was described from series of adult specimens that were relaxed with menthol crystals and fixed in dilute formalin prior to preservation in 70% ethanol. Descriptive penial terminology is from Taylor (1987) and Hershler (1994, 1998). Variation in the number of cusps on the radular teeth (*n*=5) was assessed using the method of Hershler et al. (2007).

We used a conservative, evolutionary lineage concept in describing new species only for those snails that are morphologically diagnosable as well as phylogenetically independent and substantially divergent genetically (Hershler et al. 2007). Inasmuch as the principal goal of our paper was to delimit species, we provide only brief taxonomic descriptions which focus on those aspects of morphology that have proven most useful in previous such studies of *Pyrgulopsis* (Taylor 1987, Hershler 1994, Hershler 1998).

**Table 1.** Sample codes, collection localities, and GenBank accession numbers for *Pyrgulopsis gilae* mtDNA sequences.

Code	Locality (all in New Mexico)	COI	NDI
G1	Spring along East Fork Gila River, ca. 1.53 km north, 2.9 km east of State Route 527 bridge crossing, Grant County	KC571284, KC571285	KM079175, KM079176
G2	Spring along East Fork Gila River, ca. 1.29 km north, 0.56 km west of Black Canyon confluence, Grant County	KC571286, KC571287	KM079177, KM079178
G3	Spring along East Fork Gila River, ca. 1.53 km north, 2.38 km east of State Route 527 bridge crossing, Grant County	KC571288, KC571289, KC571290	KM079176
G4	Seepage along Taylor Creek, ca. 0.32 km south, 0.93 km west of Wall Lake dam (below Wall Lake), Catron County	KC571291, KC571292, KC571293, KC571294	KM079180
G5	Hillside seep, 1.61 km north, 0.97 km east of Burnt Corral Canyon, Catron County	KC571295	KM079181
G6	Spring along Beaver Creek, ca. 0.29 km north, 0.40 km west of Taylor Creek confluence, Catron County	KC571296, KC571297, KC571298	KM079181, KM079182
G7	Seepage along Taylor Creek, 50 m west of Whitetail Canyon, Catron County	KC571292	KM079183
G8	Spring along Middle Fork Gila River, ca. 0.97 km north, 0.64 km west of Jordan Canyon, Grant County	KC571299, KC571300, KC571301, KC571302	KM079187, KM079188
G9	Fall Spring, 1.61 km north, 0.56 km east of Burnt Corral Canyon, Catron County	KC571303 KC571304	KM079184, KM079185
†G10	Fall Spring, 1.61 km north, 0.56 km east of Burnt Corral Canyon, Catron County	KC571304	KM079185, KM079186
G11	Spring along Middle Fork Gila River, ca. 0.48 km north, 0.48 km west of Jordan Canyon, Grant County	KC571305	KM079187, KM079189, KM079190
G13	Spring along Taylor Creek, 0.81 km north, 1.13 km east of Wall Lake Dam, Catron County	KC571292	KM079183
G14	“Alum Hot Spring,” ca. 1.93 km south, 0.16 km west of State Route 527 bridge crossing, Grant County	KC571288, KC571306	KM079179

†Very small (juvenile) specimens initially thought to be distinct from *Pyrgulopsis gilae*.

## Results

**Figure 2.**
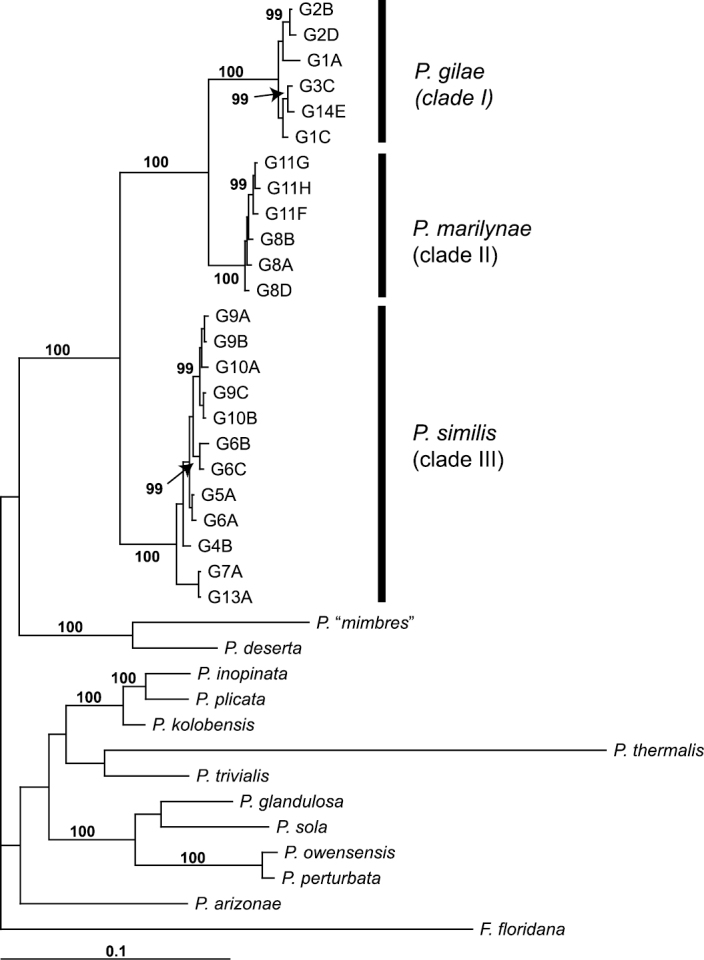
Bayesian tree based on the combined (COI, NDI) dataset. Posterior probabilities for nodes are given when >95%. Specimen codes are from the Table 1.

Sixteen (16) NDI haplotypes of *Pyrgulopsis gilae* were detected, 11 of which were restricted to single populations (Table 2). The others were shared by pairs of populations along the lower East Fork (haplotype II), upper East Fork (haplotypes VII, IX, XI) and Middle Fork (haplotype XIII) Gila River. Six (6) samples each contained a single haplotype (G3, G4, G5, G7, G13, G14). The TCS analyses (not shown) recovered three well differentiated haplotype groups composed of specimens from along the lower East Fork and main stem Gila River (clade I), Middle Fork Gila River (II), and the upper East Fork Gila River (III). These groups differed from each other by 3.7-8.7% sequence divergence; variation within groups was minor (Table 3). The AMOVA indicated that most of the detected variation (91.7%) was partitioned among these groups; variation within populations, and among populations within the groups was much smaller (1.35, 6.93%) but nonetheless was significant (Table 4). The three previously reported clades (I-III; Liu et al. 2013) were similarly recovered in Bayesian analyses of both the NDI dataset, and the combined COI + NDI dataset (Fig. 2). Based on the genetic evidence of distinctiveness and the diagnosable shell and penial characters that are detailed below we recognize two of these lineages as new species which are described herein (clade II as *Pyrgulopsis marilynae*, clade III as *Pyrgulopsis similis*) and restrict *Pyrgulopsis gilae* (clade I) to its originally circumscribed geographic range.

### Systematic descriptions
Family Hydrobiidae

Subfamily Nymphophilinae

Genus Pyrgulopsis Call and Pilsbry, 1886

#### Pyrgulopsis marilynae

Taxon classificationAnimaliaORDOHydrobiidae

Hershler, Ratcliffe, Liu, Lang and Hay sp. n.

http://zoobank.org/A641736C-650D-4649-B8AD-5A012AFB3396

Figs 3 , 4A–B

Pyrgulopsis gilae (clade II).—Liu et al. 2013.

##### Types.

Holotype, USNM 1135068 (a dry shell), spring 0.48 km north, 0.48 km west of Jordan Canyon, Catron County, New Mexico, 33.2909°N, 108.2681°W, 1 October 2009, Michelle Christman. Paratypes, USNM 1231474 (from same lot).

##### Referred material.

NEW MEXICO. *Catron County*: USNM 1123432, USNM 1123588, spring 0.8 km north, 0.64 km west of Jordan Canyon (33.2889°N, 108.2683°W), USNM 1135067, spring 0.97 km north, 0.64 km west of Jordan Canyon (33.2924°N, 108.2696°W), USNM 883175, Jordan Hot Spring (33.2927°N, 108.2692°W).

##### Diagnosis.

Distinguished from *Pyrgulopsis gilae* and the species described next (*Pyrgulopsis similis*) by its narrower shell (mean shell width/shell height 0.613 vs. 0.682, t=-9.6588, df=36.2176, *P*<0.0001, *n*=30 for *Pyrgulopsis gilae*; 0.613 vs. 0.734, t=-16.3617, df=18.9656, *P*<0.0001, *n*=11 for *Pyrgulopsis similis*), more pronounced whorl shoulders, and broad overlap of the ventral surface of the penis by the terminal gland (probably reflecting fusion with a distal ventral gland). Further differs from *Pyrgulopsis gilae* in its smaller size (mean shell height 2.77 vs. 3.47 mm, t=-11.3848, df=21.9544, *P*<0.0001) and (basal) extension of the outer penial gland to mid-line or left edge of penis. Further differs from *Pyrgulopsis similis* in its larger size (mean shell height 2.77 vs. 2.36 mm, t=7.3691, df=15.3701, *P*<0.0001), smaller number of dorsal glands on the penis, and larger size of the terminal and ventral glands on the penis.

##### Description.

**Figure 3.**
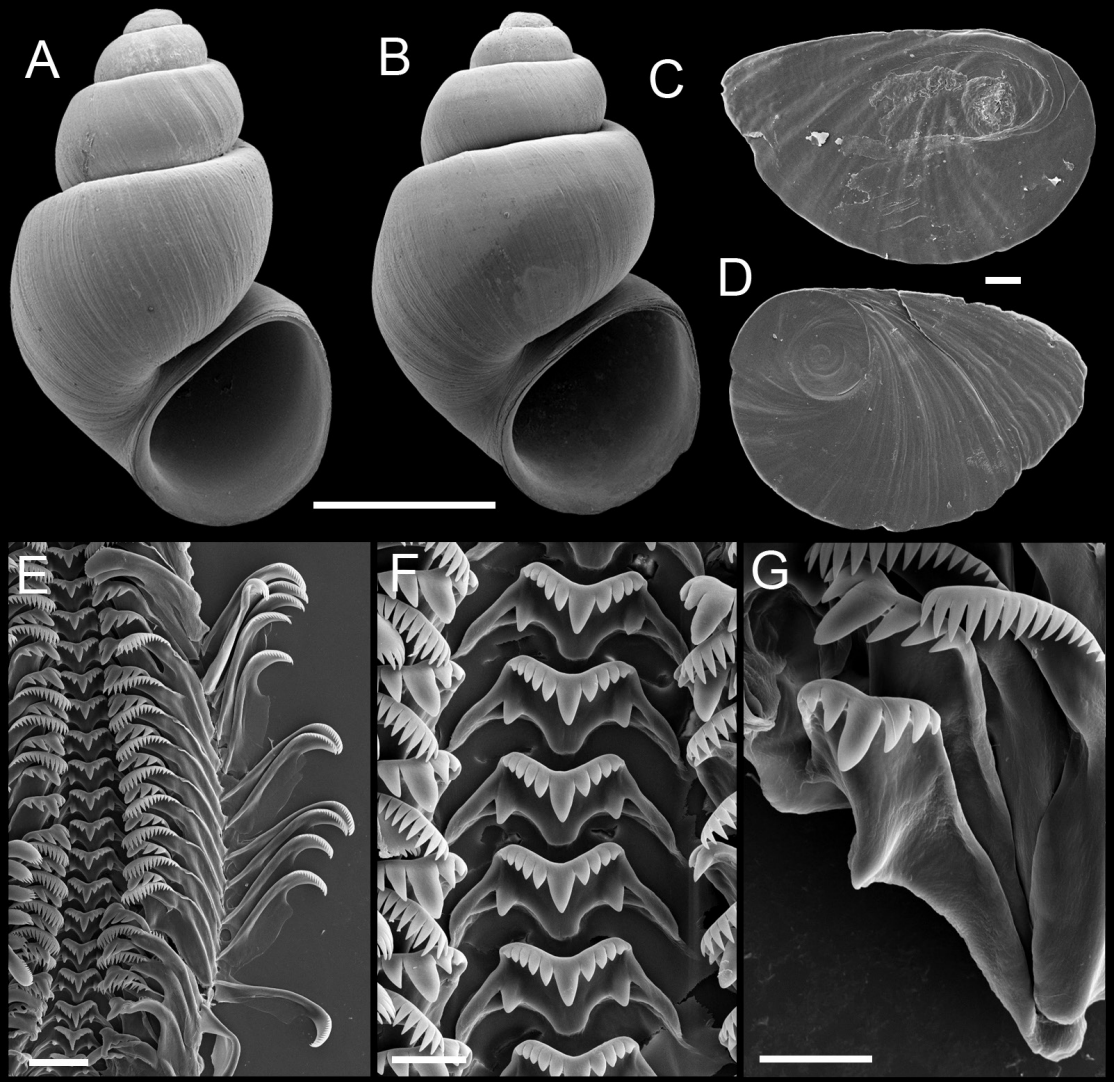
Shells, opercula and radula, *Pyrgulopsis marilynae* n. sp. **A** Holotype, USNM 1135068 **B** Shell, USNM 1135067 **C, D** Opercula (outer, inner sides), USNM 1135067 **E** Portion of radular ribbon, USNM 1135067 **F** Central teeth, USNM 1135067 **G** Lateral and inner marginal teeth, USNM 1135067. Scale bars **A, B** 1.0 mm; **C, D** 100 µm; **E** 20 µm, **F, G** 10 µm.

Shell (Fig. 3A–B) narrow-conic, whorls 4.5–5.0. Teleoconch whorls convex, shoulders narrow, angular, sutures impressed. Aperture ovate, angled above, parietal lip complete, usually slightly disjunct, umbilicus narrow. Outer lip thin, orthocline.

Operculum (Fig. 3C–D) as for genus; edges of last 0.5 whorl weakly frilled on outer side; portion of muscle attachment margin thickened on inner side. Radula (Fig. 3E–G) as for genus; dorsal edge of central teeth concave, lateral cusps four–five, basal cusp one. Lateral teeth having two–three cusps on both inner and outer sides. Inner marginal teeth with 14–20 cusps, outer marginal teeth with 17–22 cusps. Radula data are from USNM 1135067.

**Figure 4.**
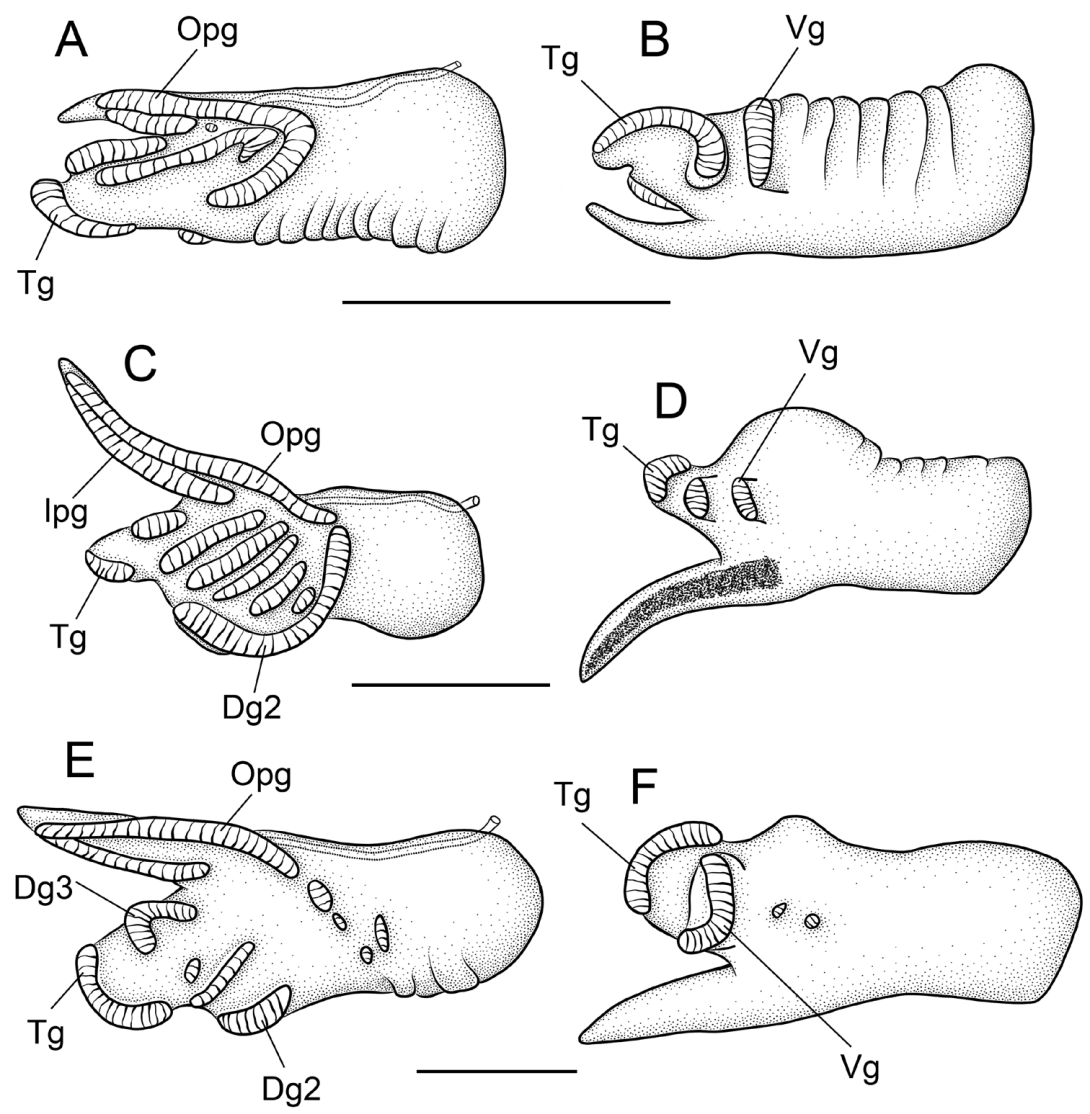
Penes (dorsal, ventral surfaces). **A, B** *Pyrgulopsis marilynae* n. sp., USNM 883175 **C, D** *Pyrgulopsis similis* n. sp., USNM 1135065 **E, F** *Pyrgulopsis gilae*, BellMNH 20898. Scale bars **A–F** 200 µm. **Dg2** dorsal gland along left edge **Dg3** dorsal gland along right distal edge **Ipg** inner (left) penial gland **Opg** outer (right) penial gland **Tg** terminal gland **Vg** ventral gland.

Penial filament and penial lobe about equal in length (Fig. 4A–B). Filament having two (penial) glands on dorsal surface; inner gland shorter. Outer penial gland curving to mid-line (10/24 specimens) or left edge of penis (14/24 specimens), the latter condition probably represents fusion with a gland on the left edge (Dg2). Terminal gland elongate, horizontal, broadly overlapping ventral surface of penis. Dorsal surface of penis having gland along right edge of lobe (Dg3) and 2-3 additional glands (22/24 specimens); one specimen did not have any additional glands and one specimen had four additional glands. Ventral gland positioned near centrally. Penial data are from USNM 1135067.

##### Etymology.

The specific epithet is a patronym honoring Marilyn Myers (United States Fish and Wildlife Service, retired) for her dedicated efforts to survey *Pyrgulopsis* habitats in the upper Gila River basin.

##### Distribution.

A series of seeps and springs along the north side of short reach (ca. 0.25 km) of the Middle Fork Gila River just below Jordan Hot Spring (Fig. 1). The type locality is a seep wall which is the lower-most occurrence of *Pyrgulopsis marilynae* along the Middle Fork Gila River; the water temperature at this site was 25°C on 1 October 2009.

##### Remarks.

*Pyrgulopsis marilyane* was resolved as sister to *Pyrgulopsis gilae* (100% posterior probability) in the molecular phylogenetic analysis (Fig. 2). The apparent fusion of the terminal and distal ventral glands of the penis that characterizes this species (in part) was previously reported for *Pyrgulopsis sadai* (Hershler 1998, fig. 39I). The sample attributed to Jordan Hot Spring (USNM 883175) may have been collected instead from a closely proximal spring as *Pyrgulopsis gilae* has not been found at the former locality during recent surveys (USFWS 2011b).

**Table 2.** Frequency distribution of NDI haplotypes detected in *Pyrgulopsis gilae*. *n*=sample size.

**Haplotype (specimen code)**	**Sample**												
	**G1**	**G2**	**G3**	**G4**	**G5**	**G6**	**G7**	**G8**	**G9**	**G10**	**G11**	**G13**	**G14**
I (G1A)	2												
II (G1C)	1		3										
III (G2B)		2											
IV (G2D)		1											
V (G14B)													4
VI (G4B)				3									
VII (G5A)					4	1							
VIII (G6B)						4							
IX (G7A)							8					4	
X (G9A)									2				
XI (G9C)									2	2			
XII (G10A)										2			
XIII (G8A)								5			1		
XIV (G8D)								1					
XV (G11F)											2		
XVI (G11H)											2		
*n*	3	3	3	3	4	5	8	6	4	4	5	4	4

**Table 3.** Mean NDI sequence divergence (maximum composite likelihood) within and among *Pyrgulopsis gilae* clades. I: G1, G2, G3, G14; II: G8, G11: III: G4, G5, G6, G7, G9 G10, G13.

	Clade I	Clade II	Clade III
Clade I	0.003+/-0.002		
Clade II	0.037+/-0.011	0.002+/-0.001	
Clade III	0.087+/-0.021	0.075+/-0.019	0.006+/-0.003

**Table 4.** Genetic differentiation among *Pyrgulopsis gilae* clades based on NDI sequences. Sub-groups=(G1, G2, G3, G14), (G8, G11), and (G4, G5, G6, G7, G9, G10, G13). Asterisked Φ values are highly significant (*P*<0.001).

Source of variation	df	variance components	% of variation	Φ statistic
Among groups	2	13.95	91.72	0.91*
Among populations within groups	10	1.05	6.93	0.84*
Within populations	43	0.21	1.35	0.99*

#### Pyrgulopsis similis

Hershler, Ratcliffe, Liu, Lang and Hay sp. n.

Figs 4C–D , 5

Pyrgulopsis gilae .—Hurt 2004 (in part; Wall Lake population).
Pyrgulopsis gilae (clade III).—Liu et al. 2013.

##### Types.

**United States:** Holotype, USNM 1135064 (a dry shell), spring along Beaver Creek, ca. 0.29 km north and 0.4 km west of confluence with Taylor Creek, Catron County, New Mexico, 33.3405°N, 108.1097°W, 21 May 2009, BKL and Marilyn Myers. Paratypes, USNM 1135065, 1231475 (from same lot).

##### Referred material.

NEW MEXICO. *Catron County*: USNM 854684, USNM 1135057, USNM 1123589, USNM 1135058, USNM 1135059, Fall Spring, 1.61 km north, 0.56 km east of Burnt Corral Canyon (33.294°N, 108.1302°W), USNM 1123590, hillside seep 1.61 km north, 0.97 km east of Burnt Corral Canyon (33.2951°N, 108.1268°W), USNM 854685, USNM 1123594, USNM 1135060, USNM 1135061, seepage along Taylor Creek, ca. 0.32 km south, 0.93 km west of Wall Lake dam (33.3457°N, 108.0904°W), USNM 854683, USNM 1123592, USNM 1135062, USNM 1135063, spring along Taylor Creek, ca. 0.81 km north, 1.13 km east of Wall Lake Dam (33.3581°N, 108.0673°W), USNM 854682, USNM 1123593, NM: Catron Co., seepage along Taylor Creek, ca. 50 m west of Whitetail Canyon (33.3613°N, 108.0576°W).

##### Diagnosis.

Differs from *Pyrgulopsis gilae* in its smaller size (mean shell height 2.36 vs. 3.47 mm, t=--22.7297, df=36.4071, *P*<0.0001, n=30 for *Pyrgulopsis gilae*), larger number of glands on the dorsal surface of the penis, frequent extension of outer penial gland and/or Dg2 to the mid-line of the penis, and smaller size of the terminal and ventral glands on the penis. Contrasted with *Pyrgulopsis similis* above.

##### Description.

**Figure 5.**
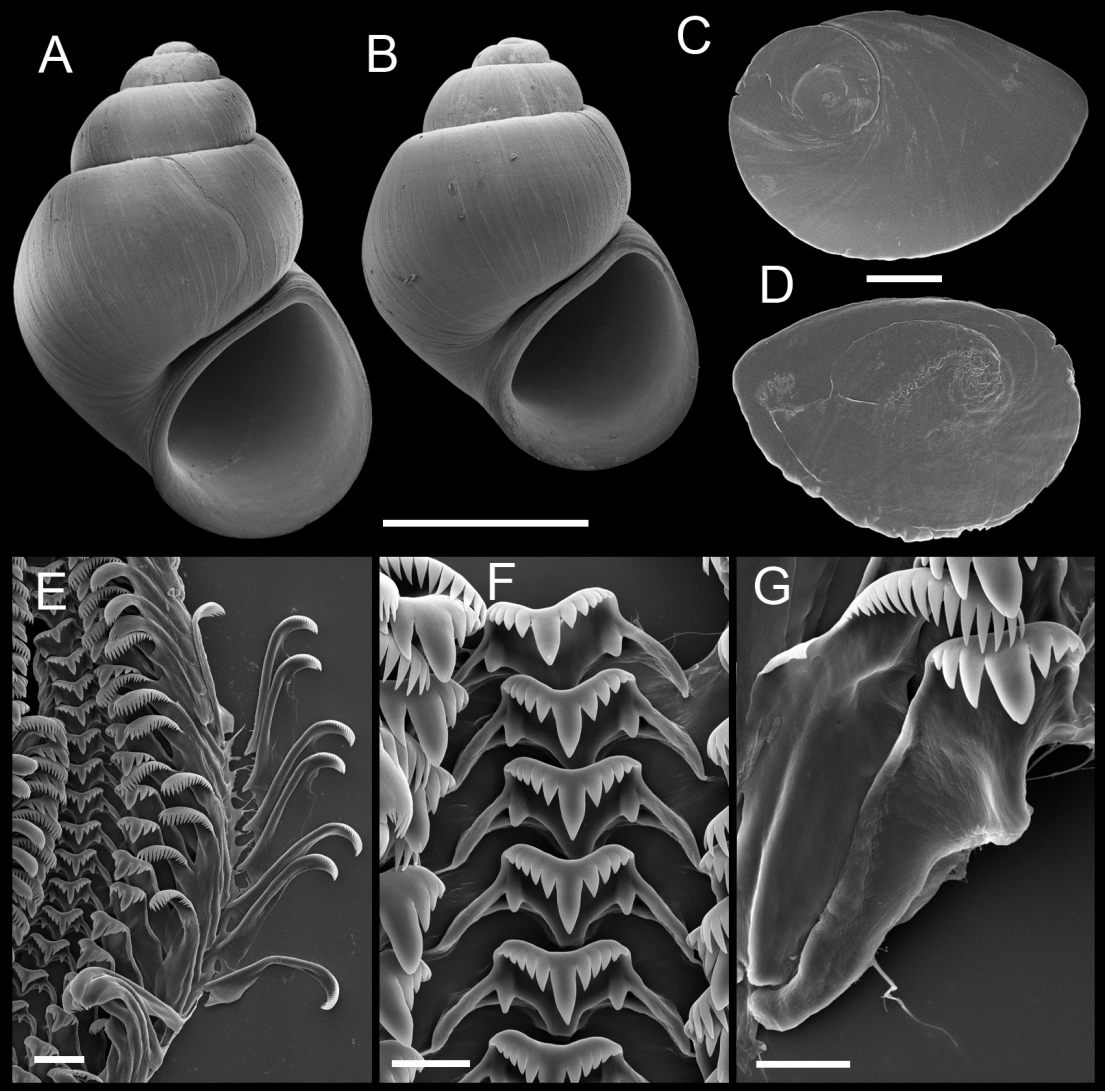
Shells, opercula and radula, *Pyrgulopsis similis* n. sp. **A** Holotype, USNM 1135064 **B** Shell, USNM 854684 **C, D** Opercula (outer, inner sides), USNM 1135065 **E** Portion of radular ribbon, USNM 1135065 **F** Central teeth, USNM 1135065 **G** Lateral and inner marginal teeth, USNM 1135065. Scale bars **A, B** 1.0 mm; **C, D** 200 µm; **E** 20 µm, **F, G** 10 µm.

Shell (Fig. 5A–B) ovate- to narrow conic, whorls 3.75–4.50. Teleoconch whorls medium convex, narrowly shouldered. Aperture pyriform, parietal lip complete, usually adnate, sometimes slightly disjunct, umbilicus small. Outer lip thin, orthocline.

Operculum (Fig. 5C–D) as for genus; edges of last 0.5 whorl frilled on outer side; inner side near smooth. Radula (Fig. 5E–G) as for genus; dorsal edge of central teeth concave, lateral cusps four–six, basal cusp one. Lateral teeth having two–three cusps on inner sides and two–four cusps on outer sides. Inner marginal teeth with 15–20 cusps, outer marginal teeth with 16–25 cusps. Radula data are from USNM 1135059, USNM 1135064.

Penial filament longer than lobe (Fig. 4C–D). Filament having two (penial) glands on dorsal surface; inner gland shorter. Outer penial gland sometimes extending (basally) to mid-line (4/30 specimens) or left edge (7/30 specimens); Dg2 sometimes curving (basally) to mid-line (11/30 specimens). Terminal gland transverse, rather small. Dorsal surface of penis having gland along right edge of lobe (Dg3) and 3-7 additional glands (30/30 specimens) which form long, slightly oblique strips. Ventral gland small, positioned near centrally; second gland rarely present (4/30 specimens). Penial data are from USNM 1135065.

##### Distribution.

Springs along a short reach (ca. 10 km) of the East Fork Gila River from just above Wall Lake to slightly above the mouth of Burnt Corral Canyon (Fig. 1). The type locality is a spring brook (ca. one m wide and 0.25 m deep) that discharges at the base of the canyon wall along the east side of Beaver Creek; the water temperature at this locality was 22.1°C on 21 May 2009. The flow at this locality is augmented by numerous small seeps.

##### Etymology.

The specific epithet is an adjective referring to the close resemblance between this species and both *Pyrgulopsis gilae* and *Pyrgulopsis marilynae*.

##### Remarks.

*Pyrgulopsis similis* was resolved as sister to the clade composed of P. *marilynae* and *Pyrgulopsis gilae* (100% posterior probability) in the Bayesian analysis of molecular data (Fig. 2).

**Table 5.** Shell parameters for *Pyrgulopsis marilynae*. Measurements are in mm.

	WH	SH	SW	HBW	WBW	AH	AW	SW/SH	HBW/SH	AH/SH
Holotype, USNM 1135068										
	4.75	2.99	1.78	2.12	1.56	1.22	1.15	0.60	0.71	0.41
USNM 12231474 (*n*=10)										
Mean	4.60	2.77	1.70	2.01	1.51	1.16	1.6	0.61	0.73	0.42
S.D.	0.13	0.15	0.08	0.10	0.07	0.05	0.05	0.01	0.02	0.0
Range	4.50–4.75	2.51–3.06	1.53–1.83	1.83–2.15	1.37–1.62	1.04–1.22	0.95–1.12	0.60–0.64	0.69–0.76	0.40–0.44

#### Pyrgulopsis gilae

(Taylor, 1987)

Fig. 4E–F

Fontelicella gilae Taylor 1987: 16-18, fig. 7, tables 11–13 (springs on north side of East Fork of Gila River, center of sec. 3, T13S, R13W, unsurveyed, Grant County, New Mexico).
Pyrgulopsis gilae .—Hershler 1994: 36–38, figs 15a–c, 46c (new combination).
Pyrgulopsis gilae .—Hurt 2004 (in part; Gila I-III populations).
Pyrgulopsis gilae (clade I).—Liu et al. 2013.

##### Types.

Holotype, LACM 2214; paratypes, BellMNH 20898, BellMNH uncat., UTEP 10054, USNM 854087 (from same lot as holotype).

##### Referred material.

NEW MEXICO. *Grant County*: USNM 1135050, USNM 1135052, spring ca. 1.29 km mile north, 0.55 km west of confluence of East Fork Gila River and Black Canyon (33.1864°N, 108.1675°W), USNSM 1123426, USNM 1135055, USNM 1135056, spring ca. 1.53 km north, 2.38 km east of State Route 527 bridge crossing (33.1946°N, 108.1804°W).

##### Other material examined.

NEW MEXICO. *Grant County*: topotypes, USNM 1004620, USNM 1135043, USNM 1135044, spring ca. 1.53 km north, 2.90 km east of State Route 527 bridge crossing (33.1917°N, 108.1742°W), BellMNH uncat., USNM 873211, USNM 1068942, “Alum Hot Spring,” ca. 1.93 km south, 0.16 km west of State Route 527 bridge crossing (33.1618°N, 108.2081°W).

##### Distribution.

Several groups of springs in the lower reach of the East Fork Gila River (below the mouth of Black Canyon) and a single spring along the Gila River ca. 2 km below the East Fork confluence (Fig. 1).

##### Remarks.

**Figure 6.**
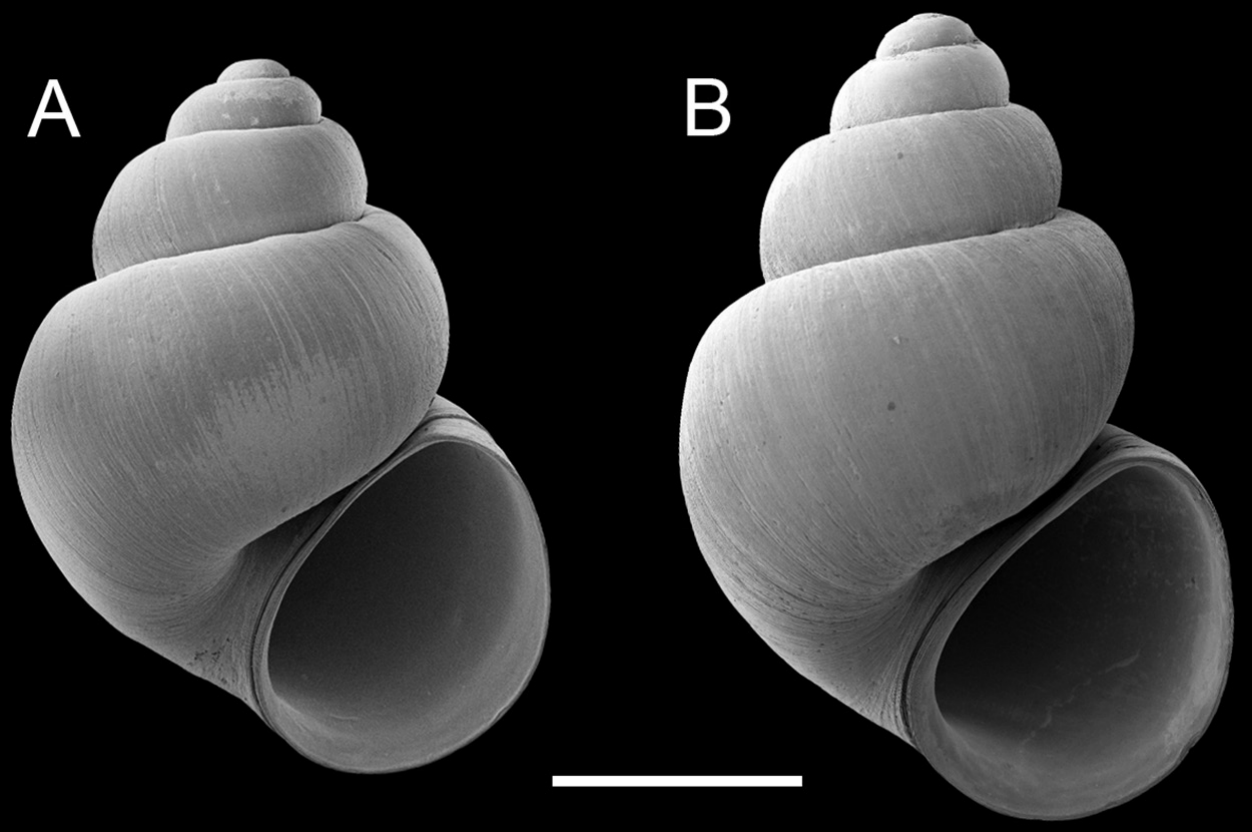
Shells, *Pyrgulopsis gilae*. **A** USNM 1135052 **B** USNM 854574. Scale bars **A, B**, 1.0 mm.

Examination of the large series of penes that Taylor scored for this species (BellMNH 20898, BellMNH uncat.) indicated that neither the outer penial gland nor Dg2 extends appreciably onto the dorsal surface of the penis (Fig. 4E–F; also see Taylor 1987, fig. 7b–c) in contrast with *Pyrgulopsis marilynae* and *Pyrgulopsis similis*. Specimens from the two new populations (Fig. 6A), which are closely proximal to the type locality, and the disjunct “Alum Spring” population (Fig. 6B) conformed to *Pyrgulopsis gilae* in all morphological details. *Pyrgulopsis gilae* co-occurs sympatrically with *Pyrgulopsis thermalis* (Taylor) at several localities (Taylor 1987).

**Table 6.** Shell parameters for *Pyrgulopsis similis*. Measurements are in mm.

	WH	SH	SW	HBW	WBW	AH	AW	SW/SH	HBW/SH	AH/SH
Holotype, USNM 1135064										
	4.25	2.52	1.73	1.94	1.48	1.24	1.10	0.69	0.77	0.49
USNM 1231475 (*n*=11)										
Mean	4.05	2.36	1.73	1.89	1.50	1.22	1.04	0.73	0.80	0.52
S.D.	0.10	0.10	0.09	0.07	0.07	0.06	0.04	0.02	0.02	0.02
Range	4.00–4.25	2.16–2.50	1.62–1.89	1.78–2.01	1.41–1.61	1.14–1.30	0.98–1.09	0.71–0.76	0.78–0.83	0.49–0.55

## Discussion

The results of this study provide additional evidence that the current taxonomy of *Pyrgulopsis* in some cases masks cryptic species diversity. Our previous revision of widely ranging *Pyrgulopsis micrococcus* revealed this taxon to be a polyphyletic composite of five species, three of which were undescribed (Hershler et al. 2013). Here we have shown that, in contrast, *Pyrgulopsis gilae* (in the broad sense) is a monophyletic species complex diagnosed by a unique penial character—the presence of two glandular strips on the dorsal surface penial filament. (Note that *Pyrgulopsis merriami* [Pilsbry and Beecher], which is distributed in an isolated basin in southeastern Nevada, has a somewhat different pattern consisting of two glands on the dorsal and one gland on the ventral surface of the filament; Hershler 1994). These disparate findings underscore the complexity of and taxonomic challenges posed by the *Pyrgulopsis* radiation, which is characterized by endemism on very fine geographic scales and extensive morphological homoplasy (Hershler 1994, Liu and Hershler 2005).

The delineation of cryptic species complexes often has important consequences for conservation (Bickford et al. 2007). *Pyrgulopsis gilae* was recently removed from the federal candidate list for listing as endangered or threatened in part owing to the discovery of populations from along the East Fork and Middle Fork of the Gila River (USFWS 2011a, USFWS 2011b) that are assigned herein to two new species. The resulting restriction of *Pyrgulopsis gilae* to its originally circumscribed geographic range—several groups of springs in the lower reach of the East Fork Gila River (below the mouth of Black Canyon) and a single spring along the Gila River ca. 2 km below the East Fork confluence—suggests the conservation status of this species should be re-visited by the USFWS. The narrow endemism of the two new species suggests that these may also merit consideration for possible listing by the USFWS.

Our findings also underscore the need for additional field surveys to further delineate the occurrences of *Pyrgulopsis* in New Mexico and to supplement the recent monograph by Taylor (1987). Large portions of the Gila River and other drainage basins in the state have yet to be carefully searched for these tiny animals.

## Supplementary Material

XML Treatment for Pyrgulopsis marilynae

XML Treatment for Pyrgulopsis similis

XML Treatment for Pyrgulopsis gilae

## Supplementary material

Supplementary material 1 Appendix 1 doi: 10.3897/zookeys.429.7865.app1 Data type: specimens data. Explanation note: GenBank accession numbers for outgroup mtDNA sequences. This dataset is made available under the Open Database License (http://opendatacommons.org/licenses/odbl/1.0/). The Open Database License (ODbL) is a license agreement intended to allow users to freely share, modify, and use this Dataset while maintaining this same freedom for others, provided that the original source and author(s) are credited.Robert Hershler, Victoria Ratcliffe, Hsiu-Ping Liu, Brian Lang, Claire Hay
