# Supplementary material for: Taxonomic revision of the Pyrgulopsis gilae (Caenogastropoda, Hydrobiidae) species complex, with descriptions of two new species from the Gila River basin, New Mexico
Source: Zookeys. 2014 Jul 30;(429):69–85. doi: 10.3897/zookeys.429.7865 (PMC4137299; doi:10.3897/zookeys.429.7865)
Supplement: Supplementary material 1 — Appendix 1 [file zookeys-429-069-s001.docx]

**Appendix 1**. GenBank accession numbers for outgroup mtDNA sequences.

| **Species** | **COI** | **NDI** |
| --- | --- | --- |
| *Pyrgulopsis arizonae* | AY627948 | AY628072 |
| *Pyrgulopsis deserta* | DQ251077 | DQ251106 |
| *Pyrgulopsis glandulosa* | AY627959 | AY628084 |
| *Pyrgulopsis inopinata* | AY426360 | AY426415 |
| *Pyrgulopsis kolobensis* | AY627939 | AY628063 |
| *Pyrgulopsis* "*mimbres*" | KM205358 | KM205359 |
| *Pyrgulopsis owensensis* | AF520922 | AY367549 |
| *Pyrgulopsis perturbata* | AY367488 | AY367550 |
| *Pyrgulopsis plicata* | AY627935 | AY628058 |
| *Pyrgulopsis sola* | AY627957 | AY628082 |
| *Pyrgulopsis thermalis* | AY627953 | AY628077 |
| *Pyrgulopsis trivialis* | AY627941 | AY628065 |
| *Floridobia floridana* | AF520916 | AY628035 |
